# Influence of the domestic COVID-19 pandemic on the pediatric emergency department

**DOI:** 10.3389/fmed.2022.941980

**Published:** 2022-08-01

**Authors:** Ying-Ju Chen, Chun-Yu Chen, En-Pei Lee, Wun-Yan Huang, Han-Ping Wu

**Affiliations:** ^1^Department of Pediatric Emergency, China Medical University Children’s Hospital, China Medical University, Taichung, Taiwan; ^2^Department of Emergency Medicine, Tungs’ Taichung MetroHarbor Hospital, Taichung, Taiwan; ^3^Division of Pediatric Critical Care Medicine, Department of Pediatrics, Chang Gung Memorial Hospital at Linko, Taoyuan, Taiwan; ^4^College of Medicine, Chang Gung University, Taoyuan, Taiwan; ^5^Department of Pediatrics, Chiayi Chang Gung Memorial Hospital, Chiayi, Taiwan

**Keywords:** pediatric, hospitalization, COVID-19, emergency, department

## Abstract

**Objectives:**

After the coronavirus disease 2019 (COVID-19) pandemic emerged, there has been a substantial decline in emergency department (ED) visits. However, the impact of the pandemic on pediatric ED (PED) visits has not been well discussed. This study aimed to compare the epidemiology and clinical characteristics of PED visits before and after the time of the COVID-19 outbreak.

**Methods:**

Data of pediatric patients admitted to the PED between February 2019 and January 2021 were retrospectively collected. All patients were divided into two groups: 1 year before the COVID-19 pandemic (group 1) and 1 year after the COVID-19 outbreak (group 2). Basic demographics, clinical characteristics, triage levels, categories of diagnosis at PED, disposition, and hospitalization rates (wards and intensive care units) were further analyzed and compared between the two groups.

**Results:**

During the study period, 48,146 pediatric patients were enrolled (30,823 in group 1, and 17,323 in group 2). PED visits represented a 43.8% annual decline. The most common diseases in the PED in group 1 were infectious diseases, whereas digestive system diseases were the most common diseases in group 2 (both *P* < 0.001). In group 2, shorter PED observational time, longer hospital stay, and higher admission rates were noted compared to those in group 1 (all *P* < 0.001).

**Conclusion:**

During the COVID-19 pandemic, the proportion of respiratory system diseases and infectious diseases sharply decreased in the PED, whereas the proportion of digestive system diseases relatively increased. The COVID-19 pandemic has impacted the nature of PED visits and we should pay more attention on digestive system diseases and the rates of out-of-hospital cardiac arrest and overall mortality.

## Introduction

The World Health Organization declared the coronavirus disease 2019 (COVID-19) outbreak, caused by severe acute respiratory syndrome coronavirus 2 (SARS-CoV-2), a pandemic on 11 March 2020 (1–3). The response team of Taiwan Centers for Disease Control (CDC) classified the COVID-19 as a Category 5 communicable disease on 15 January 2020. In the United States, children under the age of 18 years are estimated to account for 1.7% of clinical infections with SARS-CoV-2, and at least one-third of SARS-CoV-2 infections are asymptomatic (4, 5). Although the clinical findings in children with SARS-CoV-2 infection are diverse, fever and cough are the most common symptoms (6). Muscle aches, fatigue, headache, loss of taste or smell, runny nose, vomiting, and diarrhea may also be present, while pneumonia and bronchiolitis are severe clinical manifestations in children infected with SARS-CoV-2 (7). Adults infected with SARS-CoV-2 may have significant morbidity and mortality; however, severe cases are rare in children (8–10).

Global public health strategies to limit the COVID-19 pandemic include enhanced hand hygiene, physical distancing, school and business closures, wearing of face masks, and restrictions on travel and social gatherings. The impact of the COVID-19 pandemic on emergency department (ED) use in the United States was that it decreased by 42% after more than 1 month following school closure (11). A previous study that involved 210 hospitals in Japan and examined the 4 months following school closure found reductions in pediatric hospitalization for infectious diseases, ranging from 41% at the end of March to 74% at the end of May 2020 (12). In Taiwan, epidemic prevention regulations have been established, including wearing of face masks, adequate social distancing, handwashing, and use of alcohol-based rubs. However, the impact of the COVID-19 pandemic on pediatric ED (PED) visits and outcomes has not been well discussed. In this study, we aimed to compare the epidemiology and clinical characteristics of PED visits before and after the COVID-19 outbreak.

## Materials and methods

### Patient population

This retrospective observational study included all children aged 0–18 years who presented to the PED of China Medical University Children’s Hospital, from February 2019 to January 2021. All pediatric patients were divided into two groups based on the period of visit: group 1 (pre-pandemic period, February 2019 to January 2020) and group 2 (pandemic period, February 2020 to January 2021). Potentially eligible patient visits were identified by searching the China Medical University Children’s Hospital health records database. The study was approved by the Institutional Review Board of China Medical University Hospital. All methods were performed in accordance with relevant guidelines and regulations. Data were collected, reviewed, deidentified, and anonymized before analysis, and the ethics committee waived the requirement for informed consent because of the anonymized nature of the data and scientific purpose of the study.

### Study design

The following information was obtained from the medical records of each patient: age, sex, distribution of patients by month, definite diagnoses, categories of major diagnosis, disposition, initial level of triage, duration of PED stay, admission rate, length of hospital stay, intensive care unit (ICU) admission rate, length of ICU stay, mortality rate, emergency surgery, and PED revisits within 72 h. Primary discharge diagnosis codes based on the *International Classification of Diseases, tenth revision* (ICD-10) were used to categorize all patients. All patients were divided into five age groups: infant (<1 year), toddler (1 to <2 years), preschool age (2 to <7 years), school age (7–13 years), and adolescent (13–18 years). The differences in case distribution and related clinical parameters, visit presentations, and the prevalence of the six most common diagnoses between groups 1 and 2 were further analyzed and compared. The primary outcome of this study was patients who were discharged form PER and admitted to the ward or ICU, and the secondary outcome was survival to discharge from hospital.

The disease was defined based on the division of pediatric subspecialties, including infectious and parasitic diseases; neoplasm; injury and poisoning; endocrine, nutritional, metabolic, and immunity disorders; diseases of the blood and blood-forming organs; mental disorders; diseases of the nervous system and sense organs, circulatory system, respiratory system, digestive system, genitourinary system, and skin and subcutaneous tissue; congenital abnormalities; certain conditions originating in the perinatal period; symptoms, signs, and ill-defined conditions; allergy; and others (12–14). The diagnoses were divided into 17 groups based on the diagnostic classification used by many domestic researchers (15, 16).

### Statistical analysis

Categorical variables were analyzed using the chi-square test or Fisher’s exact test when appropriate. Continuous variables were analyzed using the Mann–Whitney *U* test. In the descriptive analysis, values were presented as numbers, percentages, median (IQR), and mean ± standard deviation (SD). *P*-values < 0.05 were considered statistically significant. All statistical analyses were conducted using IBM SPSS Statistics software (version 22.0; SPSS Inc., Chicago, IL, United States).

## Results

### Demographics and presentations of pediatric ED patients

During the 2-year study period, 48,146 pediatric patients presenting to the PED were enrolled in this study. Of these, 30,823 patients were categorized as group 1 (pre-pandemic period: February 2019 to January 2020) and 17,323 patients were categorized as group 2 (pandemic period: February 2020 to January 2021). In total, during the pandemic period, a 43.8% annual decline in PED visits was noted. Of all the patients, 36,604 (76%) were younger than 6 years, and 26,431 (54.9%) were males. In group 2, an increasing trend in patients younger than 2 years old (34 vs. 30%, respectively) and a decreasing trend in patients aged 2 to <7 years old (42 vs. 46%, respectively) were observed. A comparison of the demographic characteristics and presentations of children visiting the ED between Group 1 and 2 is shown in Table 1. A significant decline in children with infectious and parasitic diseases and respiratory system diseases was noted in group 2 (both *P* < 0.001). The mean length of hospitalization in group 2 was longer than that in group 1 (*P* < 0.001), whereas the duration of observation stay in the PED in group 2 was shorter than that in group 1 (*P* < 0.001). In addition, the admission and ICU admission rates in group 2 were significantly higher than those in group 1 (both *P* < 0.001). The six most common diagnostic divisions showed differences between these two groups, with the leading diagnostic division being infectious and parasitic disease in group 1 and digestive system disease in group 2 (Table 2). The incidence of influenza and hand–foot–mouth (HFM) disease significantly decreased in group 2.

**TABLE 1:** Characteristics of PED patients and presentations before and after the COVID-19 pandemic [values are n (%)].

Variables	Group 1	Group 2	*P*-value
	(*n* = 30,823)	(*n* = 17,323)	
Sex			0.481
Male	16,958 (55.0)	9,473 (54.7)	
Female	13,865 (45.0)	7,850 (45.3)	
Age			<0.001***
<1 year	4,298 (13.9)	2,824 (16.3)	<0.001***
1 to <2 years	4,992 (16.2)	3,194 (18.4)	<0.001***
2 to <7 years	14,069 (45.6)	7,227 (41.7)	<0.001***
7 to < 13 years	4,452 (14.4)	2,202 (12.7)	<0.001***
13–18 years	3,012 (9.8)	1,876 (10.8)	0.001**
Triage			<0.001***
1	347 (1.1)	300 (1.7)	<0.001***
2	7,258 (23.5)	3,786 (21.9)	<0.001***
3	21,666 (70.3)	12,132 (70.0)	0.029*
4	1,496 (4.9)	1,012 (5.8)	<0.001***
5	56 (0.2)	93 (0.5)	<0.001***
**Diagnostic divisions**			
Infectious and parasitic disease	9,941 (32.3)	4,655 (26.9)	<0.001***
Neoplasms	45 (0.2)	27 (0.2)	0.788
Injury and poisoning	455 (1.5)	374 (2.2)	<0.001***
Endocrine, nutritional, metabolic, and immunity disorders	133 (0.4)	131 (0.8)	<0.001***
Diseases of the blood and blood-forming organs	74 (0.2)	46 (0.3)	0.591
Mental disorders	79 (0.3)	46 (0.3)	0.848
Diseases of the nervous system and sense organs	985 (3.2)	711 (4.1)	<0.001***
Diseases of the circulatory system	150 (0.5)	111 (0.6)	0.027*
Diseases of the respiratory system	8,111 (26.3)	2,875 (16.6)	<0.001***
Diseases of the digestive system	7,379 (23.9)	5,764 (33.3)	<0.001***
Disease of the genitourinary system	1,228 (4.0)	994 (5.7)	<0.001***
Diseases of the skin and subcutaneous tissue	154 (0.5)	115 (0.7)	0.02*
Congenital abnormalities	69 (0.2)	49 (0.3)	0.209
Certain conditions originating in the perinatal period	213 (0.7)	137 (0.8)	0.216
Symptoms, signs, and ill-defined conditions	587 (1.9)	590 (3.4)	<0.001***
Allergy	1,180 (3.8)	685 (4.0)	0.492
Others	40 (0.1)	13 (0.1)	0.082
Hospitalization rate	5,137 (16.7)	3,750 (21.6)	<0.001***
Ward admission rate	5,024 (16.3)	3,641 (21.0)	<0.001***
ICU admission rate	113 (0.4)	109 (0.6)	<0.001***
Length of stay in hospital, median (IQR), days	3 (2)	3 (3)	<0.001***
Length of stay in ward, mean ± SD, days	3.86 ± 3.51	4.05 ± 4.01	<0.001***
Length of stay in ICU, median (IQR), days	3 (7)	3 (5)	0.85
**Mortality rate**			
OHCA at PED	8 (0.03)	8 (0.05)	0.243
IHCA	3 (0.01)	3 (0.02)	0.772
Overall mortality rate	11 (0.04)	11 (0.06)	0.171
**OHCA**			
Mortality at PED	6 (0.02)	8 (0.05)	0.099
Sustained ROSC	2 (0.006)	0 (0)	0.746
**Observation time**			
All visits at PED, median (IQR), h	2.2 (4.1)	2.2 (3.8)	<0.001***
Non-admission, median (IQR), h	1.8 (2.7)	1.8 (3)	0.009**
Admission cases, median (IQR), h	8.9 (13)	4.3 (6.7)	<0.001***
Admission cases in ward, mean ± SD, h	11.98 ± 10.23	6.16 ± 5.63	<0.001***
Admission cases in ICU, mean ± SD, h	6.32 ± 4.94	3.56 ± 3.17	<0.001***
**Referral rate**			
LMD	2,670 (8.66)	1,713 (9.89)	<0.001***
OPD	697 (2.26)	193 (1.11)	<0.001***

Student’s *t*-test, *P*-values: *P < 0.05, ** *P* < 0.01, ***P < 0.001.

Mortality rate: final = PED mortality rate + hospitalization mortality rate.

PED, pediatric emergency department; ICU, intensive care unit; OHCA, out-of-hospital cardiac arrest; IHCA, in-hospital cardiac arrest; ROSC, return of spontaneous circulation; IQR, interquartile range; LMD, local medical doctor; OPD, outpatient department.

**TABLE 2:** Common disease and five frequency diagnostic divisions during the 2020 COVID-19 pandemic period vs. the 2019 period [values are n (%)].

Diagnostic divisions	Group 1	Group 2	*P*
1. Infectious and parasitic disease	6,783 (68.23)	3,152 (67.71)	
Pharyngitis	4,008 (40.3)	2,214 (47.6)	<0.001
Tonsillitis	1,256 (12.6)	495 (10.6)	0.001
Herpangina	856 (8.6)	205 (4.4)	<0.001
HFMD	362 (3.6)	94 (2.0)	<0.001
Acute otitis media	301 (3.0)	144 (3.1)	0.769
2. Diseases of the respiratory system	6,776 (83.54)	2,215 (77.04)	
Influenza	2,821 (34.8)	102 (3.6)	<0.001
Bronchitis	1,542 (19.0)	908 (31.6)	<0.001
Pneumonia	2,116 (26.08)	850 (29.57)	<0.001
URI	235 (2.9)	162 (5.6)	<0.001
RSV bronchiolitis	62 (0.8)	193 (6.7)	<0.001
3. Diseases of the digestive system	5,848 (79.25)	5,272 (91.46)	
Acute gastritis	2,163 (29.3)	2,466 (42.8)	<0.001
Infectious colitis	1,976 (26.8)	1,466 (25.4)	<0.001
Abdomen pain	1,263 (17.1)	848 (14.7)	<0.001
Constipation	276 (3.7)	274 (4.8)	0.246
Non-infectious colitis	170 (2.3)	218 (3.8)	<0.001
4. Disease of the genitourinary system	1,100 (89.58)	910 (91.55)	
Urinary tract infection	900 (73.3)	760 (76.5)	0.318
Balanoposthitis	103 (8.4)	82 (8.3)	0.785
Orchitis and epididymitis	43 (3.5)	36 (3.6)	0.957
Hematuria	36 (2.9)	23 (2.3)	0.324
Phimosis	18 (1.5)	9 (0.9)	0.21
5. Allergy	1,180 (100)	784 (114.45)	
Urticaria	752 (63.7)	524 (76.5)	0.157
Asthma	272 (23.1)	155 (22.6)	0.084
Substance allergy	123 (10.4)	75 (11.0)	0.537
Allergic rhinitis	26 (2.2)	12 (1.8)	0.289
Anaphylaxis	7 (0.6)	18 (2.6)	0.001
6. Diseases of the nervous system	706 (71.68)	590 (82.98)	
Febrile seizure	401 (40.7)	253 (35.6)	<0.001
Headache	141 (14.3)	114 (16.0)	0.77
Epilepsy	118 (12.0)	125 (17.6)	0.04
Ear disorders	26 (2.6)	16 (2.3)	0.326
Dizziness	20 (2.0)	82 (11.5)	<0.001

HFMD, hand-foot-and-mouth disease; URI, upper respiratory tract infection; RSV, respiratory syncytial virus.

### Comparison the characteristics of patients in the different age groups between group 1 and 2

**FIGURE 1:**
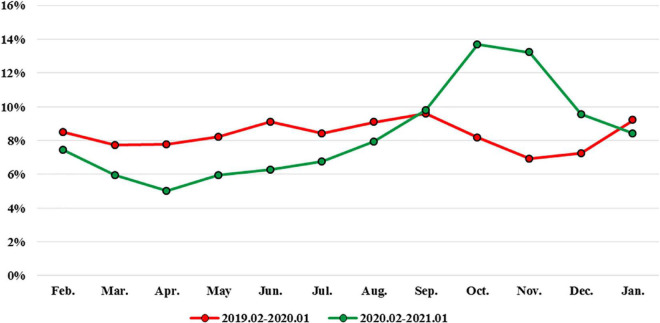
Percent change in pediatric emergency department volume vs. new Taiwan SARS-CoV-2 cases.

In Figure 1, a gradual visit rebound that started in May 2020 was noted, and visits had returned to 94% of pre-pandemic level in October 2020. We compared the epidemiology of PED visits and clinical items according to different age groups (Table 3). During the pandemic, the length of pediatric observation unit stay was shorter in all age groups, with a significant change in the infant and school-age groups (both *P* < 0.001). The rate of unscheduled return visits to the ED within 72 h also reduced after the pandemic (4.27 vs. 3.83%). However, the proportion of unscheduled return visits to the ED within 72 h in the 1–2 year age group was significantly higher after the pandemic (*P* = 0.014). The triage levels of children between the two groups were significantly different in the three age groups: 2–7 years, 7–13 years, and 13–18 years (all *P* < 0.001). In addition, the distribution of triage levels showed significant differences at 1–5 levels. Compared to that in the pre-pandemic period, the hospitalization rate of children visiting the PED significantly increased in all age groups during the pandemic period. The length of hospitalization among infants was significantly longer in group 2 than in group 1 (*P* < 0.001).

**TABLE 3:** Epidemiology of PED visits and resource use in different age groups in the 2020 pandemic period vs. the 2019 period [values are n (%)].

Variables	<1 year			1–2 years			3–6 years			7–12 years			13–18 years		
	Group 1	Group 2	*P*-value	Group 1	Group 2	*P*-value	Group 1	Group 2	*P*-value	Group 1	Group 2	*P*-value	Group 1	Group 2	*P*-value
Sex			0.042			0.299			0.064			0.133			0.006
Male	2,393 (55.68)	1,503 (53.22)		2,753 (55.15)	1,724 (53.98)		7,794 (55.4)	4,100 (56.73)		2,524 (56.69)	1,291 (58.63)		1,494 (49.6)	855 (45.58)	
Female	1,905 (44.32)	1,321 (46.78)		2,239 (44.85)	1,470 (46.02)		6,275 (44.6)	3,127 (43.27)		1,928 (43.31)	911 (41.37)		1,518 (50.4)	1,021 (54.42)	
Triage level			0.07			0.226			<0.001			<0.001			<0.001
1	44 (1.02)	44 (1.56)		53 (1.06)	46 (1.44)		178 (1.27)	157 (2.17)		42 (0.94)	31 (1.41)		30 (1)	22 (1.17)	
2	1,903 (44.28)	1,195 (42.32)		1,818 (36.42)	1,132 (35.44)		2,882 (20.48)	1,149 (15.9)		421 (9.46)	153 (6.95)		234 (7.77)	157 (8.37)	
3	2,176 (50.63)	1,445 (51.17)		2,964 (59.38)	1,893 (59.27)		10,293 (73.16)	5,435 (75.2)		3,704 (83.2)	1,839 (83.51)		2,529 (83.96)	1,520 (81.02)	
4	161 (3.75)	130 (4.6)		148 (2.96)	116 (3.63)		696 (4.95)	453 (6.27)		281 (6.31)	163 (7.4)		210 (6.97)	150 (8)	
5	14 (0.33)	10 (0.35)		9 (0.18)	7 (0.22)		20 (0.14)	33 (0.46)		4 (0.09)	16 (0.73)		9 (0.3)	27 (1.44)	
Hospitalization rate	1,574 (36.6)	1,181 (41.8)	<0.001	884 (17.7)	757 (23.7)	<0.001	1,760 (12.5)	1,148 (15.9)	<0.001	521 (11.7)	345 (15.7)	<0.001	398 (13.2)	319 (17.0)	<0.001
Ward admission rate	1,547 (36.0)	1,160 (41.1)	<0.001	871 (17.5)	742 (23.2)	<0.001	1,727 (12.3)	1,131 (15.6)	<0.001	501 (11.3)	320 (14.5)	<0.001	378 (12.5)	288 (15.3)	0.01
ICU admission rate	27 (0.6)	21 (0.7)	0.664	13 (0.3)	15 (0.5)	0.1653	33 (0.2)	17 (0.2)	1	20 (0.5)	25 (1.1)	0.002	20 (0.7)	31 (1.7)	0
Length of hospital stay, median (IQR), days	3 (2)	3 (2)	<0.001	3 (2)	3 (2)	0.065	3 (2)	3 (2)	0.153	3 (3)	3 (3)	0.650	4 (4)	4 (4)	0.91
Length of ward stay	3 (2)	3 (2)	<0.001	3 (2)	3 (2)	0.045	3 (2)	3 (2)	0.097	3 (3)	3 (3)	0.391	4 (3)	4 (3)	0.81
Length of ICU stay	3 (8)	8 (10)	0.070	5 (10)	2 (1)	0.081	3 (9)	3 (4)	0.700	2 (2)	3 (3)	0.319	3 (5)	2 (4)	0.49
**Mortality rate**															
OHCA at PED	6 (0.14)	6 (0.21)	0.463	1 (0.02)	0 (0)	1	1 (0.01)	0 (0)	1	0 (0)	2 (0.09)	0.208	0 (0)	0 (0)	N/A
IHCA	1 (0.02)	0 (0)	1	1 (0.02)	1 (0.03)	1	1 (0.01)	2 (0.03)	0.557	0 (0)	0 (0)	N/A	0 (0)	0 (0)	N/A
Overall mortality rate	7 (0.16)	6 (0.21)	0.632	2 (0.04)	1 (0.03)	1	2 (0.01)	2 (0.03)	0.88	0 (0)	2 (0.09)	0.208	0 (0)	0 (0)	N/A
**OHCA**															
Mortality at PED	6 (0.14)	6 (0.21)	0.463	1 (0.02)	0 (0)	1	1 (0.01)	0 (0)	1	0 (0)	2 (0.09)	0.208	0 (0)	0 (0)	N/A
Sustained ROSC	1 (0.02)	0 (0)	1	1 (0.02)	0 (0)	1	0 (0)	0 (0)	N/A	0 (0)	0 (0)	N/A	0 (0)	0 (0)	N/A
**Observation time (h)**															
All visits at PED, median (IQR), h	2.7 (4.9)	2.2 (3.6)	<0.001	2.9 (4.9)	3 (4)	0.081	2 (3.6)	2 (3.7)	0.001	1.8 (3.6)	1.7 (3.4)	<0.001	2.5 (4.3)	2.6 (3.9)	0.329
Non-admission, median (IQR), h	2 (3.1)	2 (2.8)	0.02	2.3 (3.2)	2.5 (3.3)	0.393	1.7 (2.3)	1.7 (2.8)	0.022	1.6 (2.3)	1.4 (2.5)	<0.001	2.1 (3.4)	2.3 (3.5)	0.719
Return visits within 72 h	172 (13.07)	99 (14.89)	0.266	296 (22.49)	183 (27.52)	0.014	620 (47.11)	290 (43.61)	0.14	142 (10.79)	55 (8.27)	0.077	86 (6.53)	38 (5.71)	0.476

PED, pediatric emergency department; ICU, intensive care unit; OHCA, out-of-hospital cardiac arrest; IHCA, in-hospital cardiac arrest; ROSC, return of spontaneous circulation; IQR, interquartile range.

### Comparison the characteristics of patients who admitted to ward and ICU between group 1 and 2

The clinical characteristics of ward admission were significantly different between groups 1 and 2 (*P* < 0.001). In group 1, infectious and parasitic diseases were the most common diseases in children admitted to the PED, followed by respiratory and digestive system diseases; however, in group 2, digestive system diseases were the most common diseases, followed by infectious and parasitic diseases and respiratory system diseases. In both groups 1 and 2, acute pharyngitis and tonsillitis were the most common diagnoses in cases of infectious and parasitic diseases, whereas acute gastritis and infectious colitis were the most common diagnoses in cases of digestive system diseases. In addition, influenza was the most common respiratory system disease in group 1, whereas acute bronchiolitis was the most common disease in group 2. The number of children with influenza dramatically decreased in group 2. Moreover, compared with group 1, the proportions of infectious and parasitic diseases and of respiratory system diseases in group 2 both showed a significant decrease (both *P* < 0.001). However, the proportions of digestive system diseases; endocrine, nutritional, metabolic, and immunity disorders; injury and poisoning; and allergy were higher in group 2 than in group 1 (all *P* < 0.05) (Table 4).

**TABLE 4:** Admission to the ward [values are n (%)].

Variables	Group 1	Group 2	*P*-value
	(*N* = 5,035)	(*N* = 3,645)	
Sex			0.798
Male	2,699 (53.6)	1,964 (53.9)	
Female	2,336 (46.4)	1,681 (46.1)	
Age			<0.001
<1 year	1,547 (30.7)	1,160 (31.8)	0.275
1 to <2 years	871 (17.3)	742 (20.4)	<0.001
2 to <7 years	1,738 (34.5)	1,135 (31.1)	0.001
7 to <13 years	501 (10)	320 (8.8)	0.066
13–18 years	378 (7.5)	288 (7.9)	0.496
Triage level			0.003
1	146 (2.9)	141 (3.9)	0.013
2	2,022 (40.2)	1,367 (37.5)	0.012
3	2,768 (55)	2,044 (56.1)	0.308
4	99 (2)	91 (2.5)	0.096
5	0 (0)	2 (0.1)	0.344
**Diagnostic divisions**			
Infectious and parasitic disease	1,342 (26.7)	847 (23.2)	<0.001
Neoplasms	42 (0.8)	17 (0.5)	0.04
Injury and poisoning	44 (0.9)	50 (1.4)	0.027
Endocrine, nutritional, metabolic, and immunity disorders	53 (1.1)	79 (2.2)	<0.001
Diseases the blood and blood-forming organs	52 (1)	34 (0.9)	0.643
Mental disorders	19 (0.4)	10 (0.3)	0.412
Diseases of the nervous system and sense organs	288 (5.7)	256 (7)	0.013
Diseases of the circulatory system	73 (1.4)	52 (1.4)	0.929
Diseases of the respiratory system	1,508 (30)	740 (20.3)	<0.001
Diseases of the digestive system	746 (14.8)	896 (24.6)	<0.001
Disease of the genitourinary system	498 (9.9)	394 (10.8)	0.164
Diseases of the skin and subcutaneous tissue	31 (0.6)	25 (0.7)	0.687
Congenital abnormalities	20 (0.4)	10 (0.3)	0.336
Certain conditions originating in the perinatal period	182 (3.6)	118 (3.2)	0.342
Symptoms, signs, and ill-defined conditions	75 (1.5)	49 (1.3)	0.574
Allergy	60 (1.2)	68 (1.9)	0.01
Others	2 (0)	0 (0)	0.626
Length of hospital stay, median (IQR), days	3 (2)	3 (2)	<0.001
Inward, mean ± SD	3.86 ± 3.51	4.05 ± 4.01	<0.001
Observation time for admission to ward, mean ± SD, h	11.98 ± 10.23	6.16 ± 5.63	<0.001

IQR, interquartile range.

**FIGURE 2:**
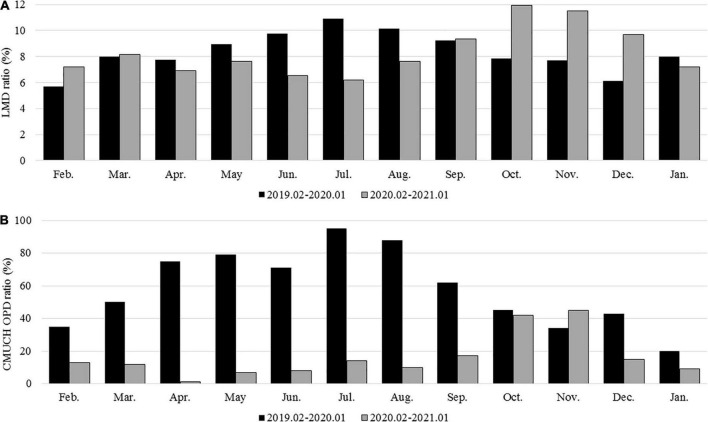
**(A)** Referred cases from local medical doctors in groups 1 and 2; **(B)** referred cases from the CMUH OPD in groups 1 and 2.

**FIGURE 3:**
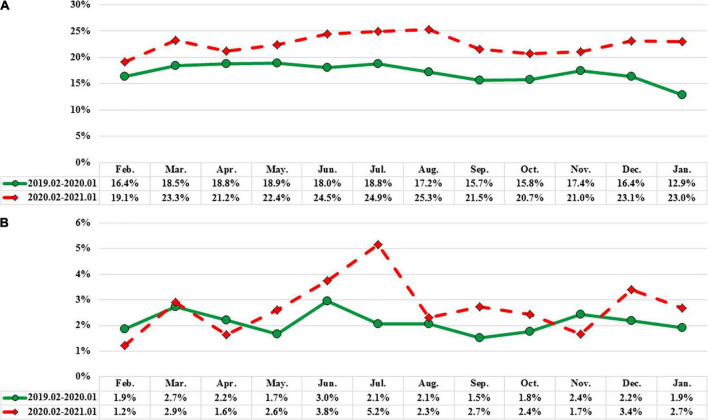
**(A)** Hospitalization ward ratio of groups 1 and 2. **(B)** Hospitalization intensive care unit ratio of groups 1 and 2.

**FIGURE 4:**
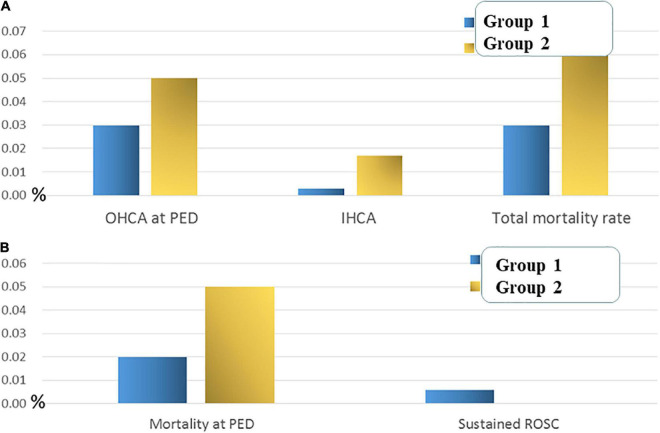
**(A)** The overall cardiac arrest rate and mortality rate of groups 1 and 2. **(B)** The mortality rate in the PED of groups 1 and 2.

The demographic characteristics of ICU admissions between groups 1 and 2 are shown in Table 5. In the ICU, infectious and parasitic diseases were significantly lower in group 2 than in group 1 (*P* < 0.05). In addition, the PED stay time at ICU admission was shorter in group 2 than in group 1 (*P* < 0.05). The rate of referrals to the PED from local medical clinics increased during the pandemic (*P* < 0.001), whereas the rate of outpatient department referral to the PED significantly declined (*P* < 0.001) (Figure 2). The admission rates of the ward and ICU for each month decreased after the pandemic (Figure 3). The mortality and out-of-hospital cardiac arrest (OHCA) rates in groups 1 and 2 are shown in Figure 4. The overall mortality and OHCA rates both increased during the pandemic period.

**TABLE 5:** Admission to the ICU [values are n (%)].

Variables	Group 1	Group 2	*P*-value
	(*N* = 113)	(*N* = 109)	
Sex			0.298
Male	74 (65.5)	64 (58.7)	
Female	39 (34.5)	45 (41.3)	
Age			0.064
<1 year	27 (23.89)	21 (19.27)	
1 to <2 year	13 (11.5)	15 (13.76)	
2 to <7 year	33 (29.2)	17 (15.6)	
7 to <13 year	20 (17.7)	25 (22.94)	
13 to <18 year	20 (17.7)	31 (28.44)	
**Triage level**			
1	36 (31.86)	37 (33.94)	0.741
2	44 (38.94)	38 (34.86)	0.529
3	33 (29.2)	34 (31.19)	0.747
**Diagnostic divisions**			
Infectious and parasitic disease	10 (8.85)	2 (1.83)	0.044
Neoplasms	1 (0.88)	5 (4.59)	0.198
Injury and poisoning	1 (0.88)	5 (4.59)	0.198
Endocrine, nutritional, metabolic, and immunity disorders	5 (4.42)	8 (7.34)	0.523
Diseases the blood and blood-forming organs	1 (0.88)	1 (0.92)	1
Mental disorders	0 (0)	0 (0)	N/A
Diseases of the nervous system and sense organs	32 (28.32)	34 (31.19)	0.748
Diseases of the circulatory system	5 (4.42)	6 (5.5)	0.951
Diseases of the respiratory system	32 (28.32)	25 (22.94)	0.445
Diseases of the digestive system	5 (4.42)	6 (5.5)	0.951
Disease of the genitourinary system	1 (0.88)	0 (0)	1
Diseases of the skin and subcutaneous tissue	0 (0)	0 (0)	N/A
Congenital abnormalities	4 (3.54)	3 (2.75)	1
Certain conditions originating in the perinatal period	5 (4.42)	2 (1.83)	0.472
Symptoms, signs, and ill-defined conditions	9 (7.96)	11 (10.09)	0.75
Allergy	2 (1.77)	1 (0.92)	1
Others	0 (0)	0 (0)	N/A
Mortality rate	3 (2.65)	3 (2.75)	0.712
Sustained OHCA at PED	2 (1.77)	0 (0)	0.493
Overall mortality rate in ICUs	3 (2.65)	3 (2.75)	0.712
PED observation time, h	6.32 ± 4.94	3.56 ± 3.17	<0.001

PED, pediatric emergency department; ICU, intensive care unit; OHCA, out-of-hospital cardiac arrest.

## Discussion

Since the COVID-19 pandemic, global public health measures have enhanced hand hygiene, physical distancing, wearing of face masks, and restrictions on travel and social gatherings. During the study period, the SARS-CoV-2 pandemic led to sharply reduced PED visits (43.8%), with especially large declines in children aged 2–6 years. Previous studies revealed that after 1–3 months, the closure of schools may lead to about 60% of PED volume decline (13–15). In this study, we also found that public health measures caused > 40% of PED visit decline. These declines were consistent with other international studies on PED visits, which showed rapid declines in ED visits and hospitalizations within 1 month of each country’s ED visits (16–18). In this study, a gradual PED visit rebound started in May 2020 and visits returned to 94% of the pre-pandemic level in October 2020.

Further, the overall estimated hospitalization rate increased to almost 21% after the pandemic, including ward admission rate from 16.1 to 21.0% and ICU admission rate from 0.4 to 0.6%. In addition, we noticed that the proportion of children admitted to the PED due to diseases of the respiratory system and infectious and parasitic diseases significantly declined after the pandemic. It has been reported that the trend of virus isolation rates of adenovirus, enterovirus, influenza virus and parainfluenza virus showed a significant reduction and RSV showed an unusual increase after COVID-19 pandemic of 2020 (19). In our present study, before the pandemic, infectious and parasitic diseases were the most common diagnoses in children admitted to the PED; however, digestive system diseases became the major diagnostic division in children visiting the PED after the pandemic. The significant decrease in the number of infectious diseases may be caused by the decline in potentially communicable diseases, such as influenza (declined by 95.5%) and HFM disease (declined by 74%). Regarding respiratory system diseases, influenza significantly changed before and after the pandemic and almost disappeared after the pandemic. In addition, HFM disease and herpangina caused by enterovirus infection significantly decreased by three quarters after the pandemic. These diseases are often caused by droplet induction and contact infections. However, after the pandemic, public health policies such as enhanced hand hygiene, physical distancing, wearing of face masks, and restrictions on travel and social gatherings have been promoted and implemented to avoid COVID-19 infection, which has effectively decreased relative diseases, including influenza, HFM disease, and herpangina. In addition, to mitigate the transmission of enterovirus in children, infected children ≤8 years are asked to stay at home for 7 days in Taiwan (20).

Diseases of the digestive system seem to have declined relatively less after the pandemic in central Taiwan. It has been reported that intense non-pharmaceutical interventions to stop COVID-19 transmissions, such as social distancing and school closure may have a positive effect on the control of norovirus-related acute gastroenteritis (21). During the COVID-19 pandemic period 2020, we did not close schools and parents and schools may focus less attention on gastrointestinal infectious disease. These may lead to transmit gastrointestinal infectious disease and initiate outbreaks at home and at school. Public health strategies play an important role not only in social restrictions and the personal prevention of SARS-CoV-2 but also in health education and medical system response. In addition, we must pay more attention to the prevention and treatment of digestive system diseases.

Overall, 3,645 children were admitted to the pediatric ward during the study period of group 2, compared to 5,035 ward admissions recorded during the study period of group 1 (−27.6%). However, higher ward and ICU admission rates were noted after the COVID-19 pandemic. It has been reported that the proportion of those admitted to hospital was strongly associated with the triage level (22). In our present study, the proportion of children triaged as level 1 showed significantly increased and level 2 and 3 showed significantly decreased after the COVID-19 pandemic. The overall ward admission rate increased after the COVID-19 pandemic may indicate that the characteristics of disease pattern and the severity of PED visits may be changed and children triaged as level 2 and 3 should evaluate more carefully than before. Hospitalized pediatric patients had a longer length of hospital stay after the pandemic compared to that before the pandemic; however, the length of observation time in the PED was significantly shorter after the COVID-19 pandemic. This may indicate that changes in visit characteristics in the PED and early discharge from the ED with shortened PED observation time for patients’ families may reduce the risk of SARS-CoV-2 infection during ED visits. This finding may reflect the differing impacts of pandemic restrictions on children with different diseases and different ages admitted to the PED. Moreover, this may also cause some changes in the healthcare-seeking behaviors of patients and families. Although the number of children with OHCA does not show a significant difference between the periods before and after the pandemic, the OHCA rate and overall mortality both increased during the pandemic. This indicates that emergency and critical care for pediatric patients with OHCA should be promoted and updated to rescue critical cases admitted to PEDs and ICUs, even during the pandemic.

The present study had some limitations. First, in a retrospective single-center review of medical records, some details of the prognosis may not be rigorously documented. Second, our data included only one PED in the children’s hospital in central Taiwan; other ED settings in other areas may present differences in patient presentation patterns of ED.

## Conclusion

In the PED, the proportion of respiratory system diseases and infectious diseases sharply decreased during the COVID-19 pandemic, whereas the proportion of digestive system diseases relatively increased. Higher ward and ICU admission rates with increased length of hospital stay were noted during the 1-year pandemic period. In addition, the trend of OHCA rate and overall mortality seems to both increased during the pandemic.

## Data availability statement

The original contributions presented in this study are included in the article/supplementary material, further inquiries can be directed to the corresponding author.

## Ethics statement

The study was approved by the Institutional Review Board of China Medical University Hospital. Written informed consent for participation was not provided by the participants’ legal guardians/next of kin because the ethics committee waived the requirement for informed consent because of the anonymized nature of the data and scientific purpose of the study.

## Author contributions

C-YC and Y-JC reviewed the medical records, analyzed and interpreted the data, drafted the manuscript, and reviewed the medical. E-PL and W-YH analyzed and interpreted the data. H-PW and C-YC designed and oversaw the study and revised the manuscript. All authors contributed to the article and approved the submitted version.

## Conflict of interest

The authors declare that the research was conducted in the absence of any commercial or financial relationships that could be construed as a potential conflict of interest.

## Publisher’s note

All claims expressed in this article are solely those of the authors and do not necessarily represent those of their affiliated organizations, or those of the publisher, the editors and the reviewers. Any product that may be evaluated in this article, or claim that may be made by its manufacturer, is not guaranteed or endorsed by the publisher.

## Abbreviations

COVID-19: coronavirus disease 2019
ED: emergency department
PED: pediatric ED
SARS-CoV-2: Severe acute respiratory syndrome coronavirus 2
CDC: Centers for Disease Control
ICU: intensive care unit
OHCA: out-of-hospital cardiac arrest.

## References

[1] Macchi J Herskovitz J Senan AM Dutta D Nath B Oleynikov MD The natural history, pathobiology, and clinical manifestations of SARS-CoV-2 infections. *J Neuroimmune Pharmacol.* (2020) 15:359–86. 10.1007/s11481-020-09944-5 32696264 PMC7373339

[2] Zhou P Yang XL Wang XG Hu B Zhang L Zhang W A pneumonia outbreak associated with a new coronavirus of probable bat origin. *Nature.* (2020) 579:270–3. 32015507 10.1038/s41586-020-2012-7 PMC7095418

[3] World Health Organization. *Archived: WHO Timeline – COVID-19.* Geneva: World Health Organization (2020).

[4] Oran DP Topol EJ. The proportion of SARS-CoV-2 infections that are asymptomatic: a systematic review. *Ann Intern Med.* (2021) 174:655–62. 33481642 10.7326/M20-6976 PMC7839426

[5] Irfan O Muttalib F Tang K Jiang L Lassi ZS Bhutta Z. Clinical characteristics, treatment and outcomes of paediatric COVID-19: a systematic review and meta-analysis. *Arch Dis Child.* (2021) 106:440–8. 10.1136/archdischild-2020-321385 PMC8070630 33593743

[6] Darmawan DO Gwal K Goudy BD Jhawar S Nandalike K. Vaping in today’s pandemic: e-cigarette, or vaping, product use-associated lung injury mimicking COVID-19 in teenagers presenting with respiratory distress. *SAGE Open Med Case Rep.* (2020) 8:2050313X20969590. 10.1177/2050313X20969590 33194204 PMC7607755

[7] Ludvigsson JF. Systematic review of COVID-19 in children shows milder cases and better prognosis than adults. *Acta Paediatr.* (2020) 109:1088–95. 32202343 10.1111/apa.15270 PMC7228328

[8] Brodin P. Why is COVID-19 so mild in children? *Acta Paediatr.* (2020) 109:1082–3. 32212348 10.1111/apa.15271

[9] Livingston E Bucher K. Coronavirus disease 2019 (COVID-19) in Italy. *JAMA.* (2020) 323:1335. 32181795 10.1001/jama.2020.4344

[10] Richardson S Hirsch JS Narasimhan M Crawford JM McGinn T Davidson KW Presenting characteristics, comorbidities, and outcomes among 5700 patients hospitalized with COVID-19 in the New York city area. *JAMA.* (2020) 323:2052–9. 10.1001/jama.2020.6775 32320003 PMC7177629

[11] Hartnett KP Kite-Powell A DeVies J Coletta MA Boehmer TK Adjemian J Impact of the COVID-19 pandemic on emergency department visits – United States, January 1, 2019 – May 30, 2020. *MMWR Morb Mortal Wkly Rep.* (2020) 69:699–704. 32525856 10.15585/mmwr.mm6923e1 PMC7315789

[12] Kishimoto K Bun S Shin JH Takada D Morishita T Kunisawa S Early impact of school closure and social distancing for COVID-19 on the number of inpatients with childhood non-COVID-19 acute infections in Japan. *Eur J Pediatr.* (2021) 180:2871–8. 10.1007/s00431-021-04043-w 33791861 PMC8012019

[13] Pines JM Zocchi MS Black BS Carlson JN Celedon P Moghtaderi A Characterizing pediatric emergency department visits during the COVID-19 pandemic. *Am J Emerg Med.* (2021) 41:201–4. 33257144 10.1016/j.ajem.2020.11.037 PMC7682424

[14] Westgard BC Morgan MW Vazquez-Benitez G Erickson LO Zwank MD. An analysis of changes in emergency department visits after a state declaration during the time of COVID-19. *Ann Emerg Med.* (2020) 76:595–601. 33008651 10.1016/j.annemergmed.2020.06.019 PMC7287464

[15] Todd IMF Miller JE Rowe SL Burgner DP Sullivan SG. Changes in infection-related hospitalizations in children following pandemic restrictions: an interrupted time-series analysis of total population data. *Int J Epidemiol.* (2021) 50:1435–43. 10.1093/ije/dyab101 34056664 PMC8195105

[16] Angoulvant F Ouldali N Yang DD Filser M Gajdos V Rybak A COVID-19 pandemic: impact caused by school closure and national lockdown on pediatric visits and admissions for viral and nonviral infections–a time series analysis. *Clin Infect Dis.* (2021) 72:319–22. 10.1093/cid/ciaa710 33501967 PMC7314162

[17] Dopfer C Wetzke M Scharff AZ Mueller F Dressler F Baumann U COVID-19 related reduction in pediatric emergency healthcare utilization – a concerning trend. *BMC Pediatr.* (2020) 20:427. 10.1186/s12887-020-02303-6 32894080 PMC7475725

[18] Ciofi Degli Atti ML Campana A Muda AO Concato C Ravà L Ricotta L Facing SARS-CoV-2 pandemic at a COVID-19 regional children’s hospital in Italy. *Pediatr Infect Dis J.* (2020) 39:e221–5. 10.1097/INF.0000000000002811 32639459

[19] Hsu HT Huang FL Ting PJ Chang CC Chen PY. The epidemiological features of pediatric viral respiratory infection during the COVID-19 pandemic in Taiwan. *J Microbiol Immunol Infect.* (2021). 9:S1684–1182(21)00205-X. 10.1016/j.jmii.2021.09.017 [Epub ahead of print]. 34756671 PMC8501510

[20] Lee PI Tsai TC Huang YC Wu CF Hu YL Lin TY. Effectiveness of case isolation and class suspension in mitigation of enterovirus transmission in children. *J Infect Public Health.* (2022) 15:594–8. 10.1016/j.jiph.2022.04.010 35500544

[21] Lu Y Zhang Z Xie H Su W Wang H Wang D The rise in norovirus-related acute gastroenteritis during the fight against the COVID-19 pandemic in Southern China. *Front Public Health.* (2022) 9:785373. 10.3389/fpubh.2021.785373 35087785 PMC8787315

[22] Gravel J Manzano S Arsenault M. Validity of the Canadian paediatric triage and acuity scale in a tertiary care hospital. *CJEM.* (2009) 11:23–8. 10.1017/s1481803500010885 19166636

